# Recent progress of non-linear topological structure polymers: synthesis, and gene delivery

**DOI:** 10.1186/s12951-024-02299-6

**Published:** 2024-01-27

**Authors:** Chenfei Wang, Wei He, Feifei Wang, Haiyang Yong, Tao Bo, Dingjin Yao, Yitong Zhao, Chaolan Pan, Qiaoyu Cao, Si Zhang, Ming Li

**Affiliations:** 1 Department of Dermatology, Children’s Hospital of Fudan University, National Children’s Medical Center, Shanghai, 201102 China; 2 School of Medicine, Anhui University of Science and Technology, Huainan, 232000 Anhui China; 3 Department of Anesthesiology, The Second Affiliated Hospital of Air Force Medical University, Xi’an, 710032 Shaanxi China; 4 School of Chemical Engineering and Technology, Xi’an Jiaotong University, Xi’an, 710049 Shaanxi China; 5 Key Laboratory of Glycoconjugate Research Ministry of Public Health, Department of Biochemistry and Molecular Biology, School of Basic Medical Sciences, Fudan University, Shanghai, 200032 China; 6 Shanghai EditorGene Technology Co., Ltd, Shanghai, 200000 China

**Keywords:** Gene delivery, Transfection performance, Non-viral gene vectors, Non-linear topological structure polymers, Synthetic approaches

## Abstract

Currently, many types of non-linear topological structure polymers, such as brush-shaped, star, branched and dendritic structures, have captured much attention in the field of gene delivery and nanomedicine. Compared with linear polymers, non-linear topological structural polymers offer many advantages, including multiple terminal groups, broad and complicated spatial architecture and multi-functionality sites to enhance gene delivery efficiency and targeting capabilities. Nevertheless, the complexity of their synthesis process severely hampers the development and applications of nonlinear topological polymers. This review aims to highlight various synthetic approaches of non-linear topological architecture polymers, including reversible-deactivation radical polymerization (RDRP) including atom-transfer radical polymerization (ATRP), nitroxide-mediated polymerization (NMP), reversible addition-fragmentation chain transfer (RAFT) polymerization, click chemistry reactions and Michael addition, and thoroughly discuss their advantages and disadvantages, as well as analyze their further application potential. Finally, we comprehensively discuss and summarize different non-linear topological structure polymers for genetic materials delivering performance both in vitro and in vivo, which indicated that topological effects and nonlinear topologies play a crucial role in enhancing the transfection performance of polymeric vectors. This review offered a promising guideline for the design and development of novel nonlinear polymers and facilitated the development of a new generation of polymer-based gene vectors.

## Introduction

Gene therapy holds great promise for the treatment of various inherited and acquired diseases by delivering functional genes into targeted cells or tissues to elicit manipulation of gene expression [1, 2]. Therefore, safe and efficient gene vectors are critical for the development of gene therapy [3]. Owing to its tailorable composition, topological structure and molecular weight (MW), biodegradability, and biocompatibility, polymer, as a promising clinically advanced non-viral vector, has been employed to facilitate the delivery of DNA, RNA, and protein [4–12]. In addition to monomer composition and MW, topological structure has significant effects on the gene transfection performance of cationic polymers. Notably, compared to linear poly(ethylene imine), the cyclic poly(ethylene imine) exhibited a more compact assembly structure, smaller gyration radius and higher transfection efficiency [13]. The brush polymer with more side chains, exhibited higher DNA condensation and gene transfection efficiency [14]. Star polymers tend to encapsulate genes in their cores, efficiently controlling gene release into the cytosol [15–17]. The high-density polyethylene, as a stiff polymer, was used for drainage pipes, whereas branched polyethylene with better flexibility exhibited superior packaging properties [18]. Therefore, it is imperative to explore the relationship between the topological structure and the properties/applications of polymer.

To date, a variety of non-linear topological polymers such as brush, star, highly branched structure, dendritic, cyclic and multicyclic structural polymers have been successfully synthesized. Notably, for the non-linear topological polymers, many synthetic methods including reversible-deactivation radical polymerization (RDRP) such as atom-transfer radical polymerization (ATRP), reversible addition-fragmentation chain transfer (RAFT) polymerization and nitroxide-mediated polymerization (NMP) [19–21], click chemistry reactions [22], Michael addition [23] have been proposed. However, there are still many challenges for the synthesis of non-linear topological polymers, including (1) the precise synthesis of polymers with specific topology and low polymer dispersity index (PDI); (2) the simple and efficient separation of different topological polymers according to the differences in structure and properties; (3) the large-scale synthesis of non-linear topological polymers such as dendritic polymers [24]. Thus, it is essential to comprehensively summarized the main synthetic methods of non-linear topological polymers, which provide a valuable platform for the design and development of versatile polymers.

Polymer self-assemblers represents one of the most studied nanocarriers for delivery systems [25, 26]. Nevertheless, previous investigations have suggested that the topological architecture exerts a notable influence on the assembled structure of polymers. Hyperbranched structures, star-shaped structures and cyclic structures tend to increase the stability of polymer assemblies. Therefore, various non-linear topological polymers could have potential in the hydrophobic drug delivery, various genetic materials and protein delivery [27]. For example, star-shaped polymers can encapsulate genes within their cores, thereby promoting the sustained release of the genetic material [15–17]. Due to their multiple terminal groups and three-dimensional topology, Highly branched polymers exhibited higher gene affinity and gene transfection efficiency [28–30]. Dendrimer offers a precise topology and MW, which is conducive to precision and personalized therapy [31–33]. Due to its more compact assembly structure, the cyclic polymer demonstrated high gene condensation and transfection efficiency [13, 34]. Therefore, it is expected that this review will elaborately introduce the current status and research progress of various non-linear topological polymers for gene delivery, and may advance design high performance non-linear polymeric gene vehicles for clinical trials.

## Preparation of various non-linear topological structure polymers

Due to the poor mechanical properties and thermal stability of linear polymers, cross-linking reactions are usually used to create polymer networks with high mechanical properties, such as elasticity, thermal stability, and porosity. Yet, cross-linked network polymers were mainly used as substrates for strength engineering materials. The polymer with network structure often has ultra-high MW, which makes it difficult to dissolve and melt [35, 36]. There are many uncertain defects in the network polymer, such as cyclic roop and uncross-linked chains, which cause the difference in performance and structure. More importantly, previous investigations have demonstrated that brush structures, hyperbranched structures, star-shaped structures or cyclic structures tend to improve their stability and assembly [37–39]. Therefore, it is crucial to understand how topological structure influences their transfection properties. This chapter will mainly discuss the synthesis methods of non-linear topology polymers including brush, star, and hyperbranched structures.

### Preparation of brush polymers

**Fig. 1:**
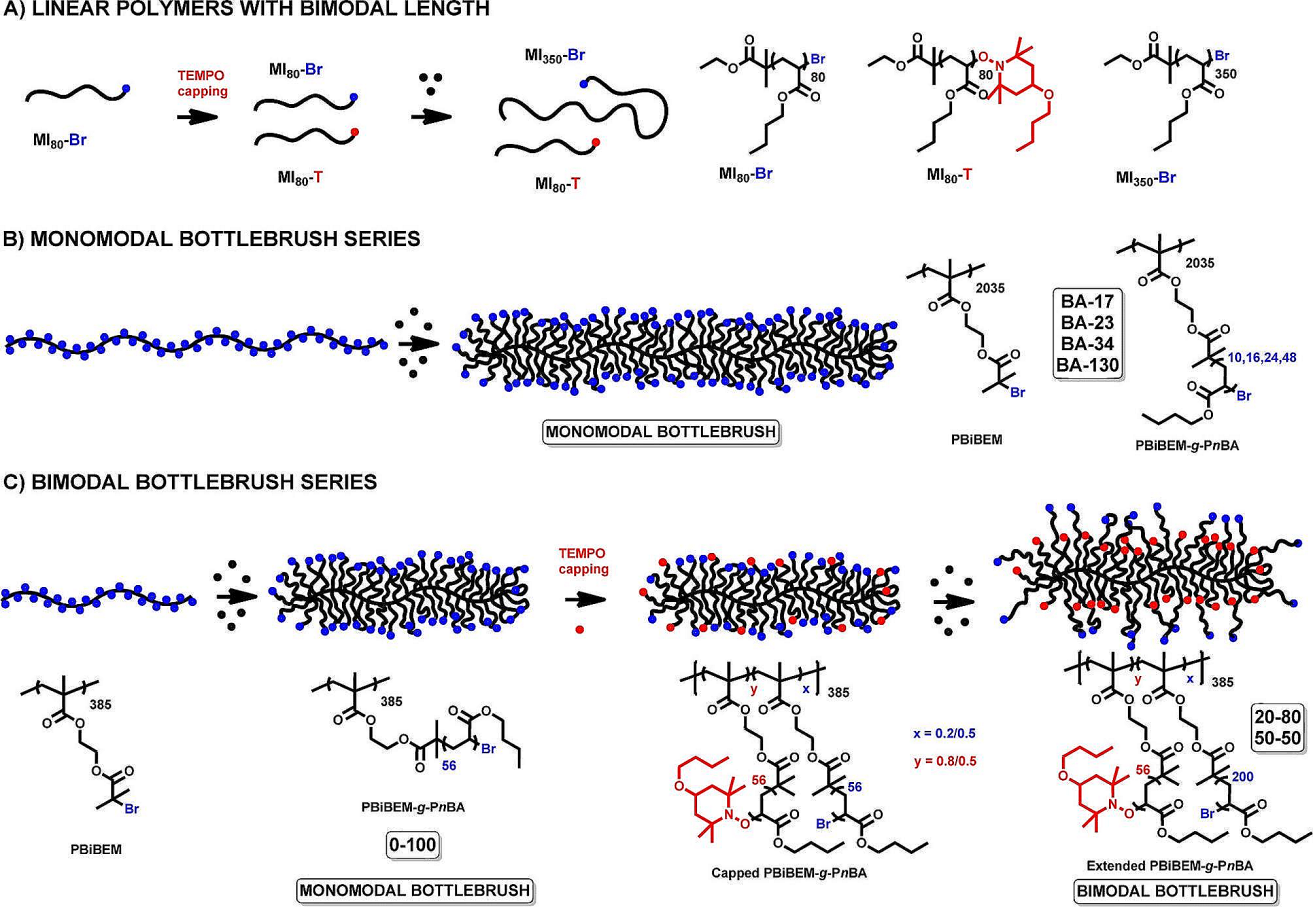
Preparation of brush polymers *via* ATRP [50]

Brush-like polymer, as a novel branched structure polymer, is formed by grafting different short side chain molecules onto the backbone chain [40, 41]. The physical and chemical properties of brush-like polymers are mainly affected by three main components [42]. Firstly, duo to large number of pendant chains brush-like polymers endowed multiple functionalization sites. Secondly, the chain entanglement of the brush-like polymer could be avoided owing to the spatial mutual exclusion of the side chains. Thirdly, by adjusting the density of the brush and the functionalized short-chain groups, the optimized brush-like polymer can meet different applications, including gene delivery, multi-functional stimuli-responsive polymers, and lubrication [43–45]. In general, the synthesis of the brush-like macromolecules included three methods, “grafting-through” (the brush-like polymer is polymerized *via* macromonomers polymerization), “grafting-to” (the brush-like polymer is prepared *via* grafting the side chain integrally to a backbone), and “grafting-from” (the brush-like polymer is constructed through monomers polymerization from a backbone) [45–47]. For the grafting-through method free radical polymerization was used, the controllability of the main chain length is generally poor. The grafting-to method, utilized click chemistry reactions, is employed to improve grafting efficiency [48]. The slow diffusion of the side chains and the hindrance of their interaction ultimately resulted in lower graft density. For grafting-from method, the reaction site is pre-set on the main chain to precisely regulate the density and the length of the side chain, employing RDRP. Therefore, this method has emerged as a conventional synthetic route for fabricating brush polymers [49]. Burdynska et al. utilized the “grafting-from” method to prepare a bottle-brush polymer with both shorter and longer grafted chains and narrow molecular weight distribution (MWD) (Fig. 1) [50]. Meanwhile, using atomic force microscope (AFM) and the Langmuir–Blodgett technique, the worm-like topology with high graft density was wrapped by loose side chains, which further confirmed the bimodal topology of the bottle brush polymer and prepared a zwitterionic bottlebrush polymers [51].

Alper et al. grafted fluorescein O-methacrylate onto the side chain of brush polymer through ATRP to prepare a pH-responsive fluorescent brush polymer, which displayed obvious fluoresce intensity properties under basic conditions, but no fluorescence under neutral or acidic conditions [52]. Due to the repulsion between the side chains, the main chain is stretched or even broken, Li et al. designed a dithiothreitol brush polymer, and proved that the inherent driving force exerting a notable influence on the diminishment and cleavage of the disulfide linkage within brush polymers [53]. This data further indicated that the number of side chains affects its main chain scission and strength [54]. Therefore, brush polymer holds great promise in the degradable materials, controlled release and self-healing materials. In addition, during ATRP process, the partial linkage between the chain ends caused the MW of polymer to increase rapidly, and appeared to gel. To address these issues, Xie et al. used acrylate monomers through ATRP under catalytic free radical termination (CRT) to prepare brush polymers with high side chain density at high monomer conversion, and effectively inhibiting gelation [55]. Chen et al. used ring-opening polymerization (ROP), click chemistry reaction combined with ATRP to prepare a new brush-on-brush architecture polymer with poly(oligo(ethylene glycol) acrylate) brush. Brush-on-brush architecture polymers exhibited more side chains, which could increase the density and dimensionality of side chains to overcome the repulsion between side chains [56]. AFM proved wormlike formation and multi-level structure brushes. The hydrophilicity and hydrophobicity of brush polymers were regulated through similar approach, which could greatly expand the brush polymers in drug/gene delivery applications [57]. Moreover, as an alternative, RAFT polymerization is a promising technique that can potentially replace ATRP to avoid toxic metal copper as a catalyst for brush polymer preparation. The atom transfer radical polyaddition (ATRPA), as a new method to prepare aliphatic polyester, also has attracted many attentions [58]. Yang et al. used ATRPA to prepare the linear poly(4-vinylbenzyl 2-bromo-2-phenylacetate), and then prepared poly(N-vinylpyrrolidone) side chains to construct new bottle brush polymers. This study cleverly combined two controllable living polymerization methods to prepare brush polymers.

In the process of ATRP polymerization, metal copper ions, as a catalyst, are indispensable. However, the complicated and costly separation process greatly hinders the synthesis of brush polymers. Lai et al. proposed metal-free catalyzed RAFT polymerization and ROP to prepare bottle brush macromolecules, effectively avoiding the use of metal copper catalysts [59].

**Fig. 2:**
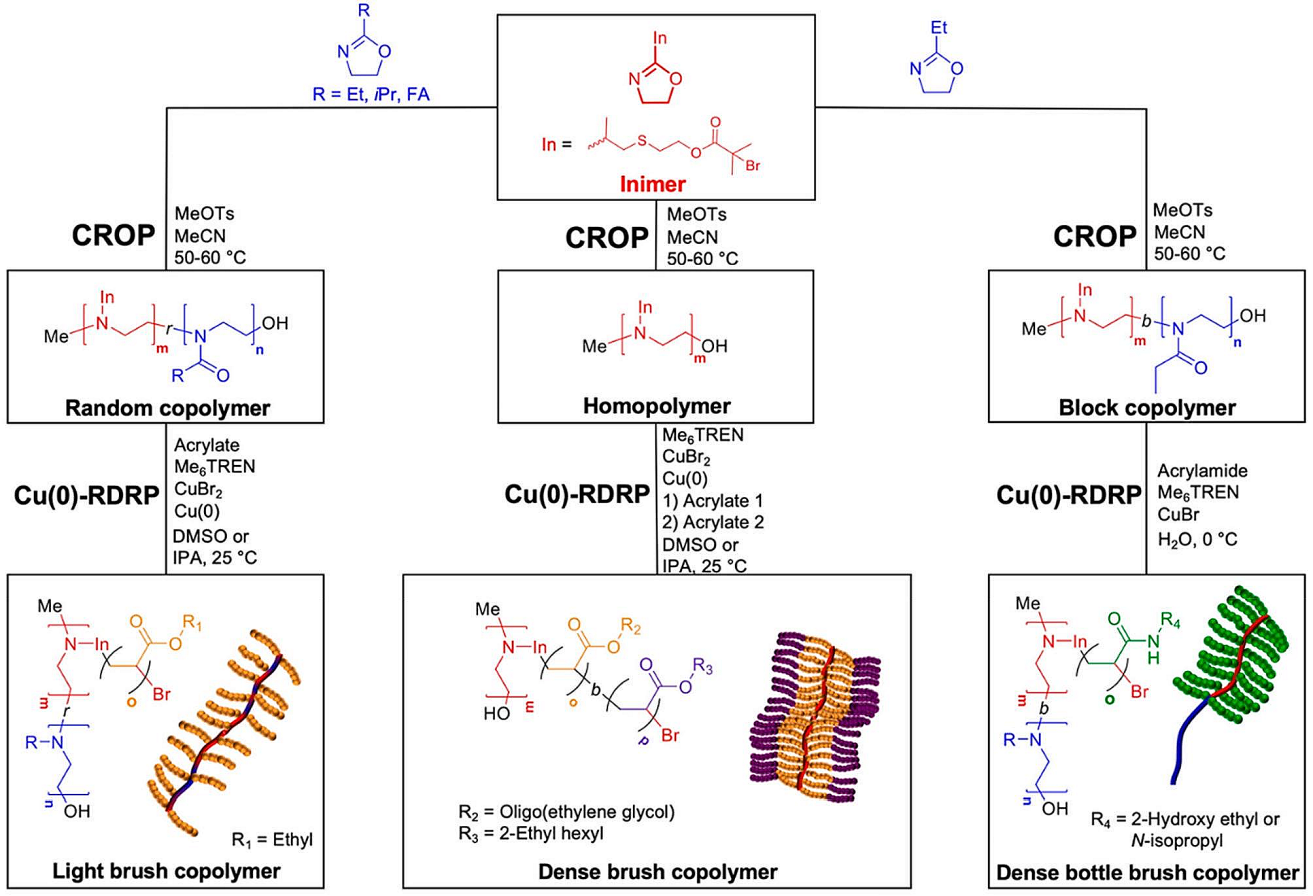
Synthesis of molecular bottlebrushes *via* cationic ring-opening and RAFT polymerization [60]

Alentin et al. used 2-oxazoline as monomer to prepare various types of linear backbones through cationic ROP. Then acrylates and acrylamides are polymerized to prepare three types of brush polymers including low/high density brush, bottle brush [60], detailed synthesis process shown in Fig. 2. This research ingeniously designs various types of brush polymers utilizing random copolymerization, homopolymerization, and block polymerization. 2-Oxazoline and acrylate/acrylamide firstly were used to prepare brush polymers. Furthermore, this reaction was easily scaled up to several dozen g scale. Su et al. used phenyl isocyanide monomer to prepare a brush-like polymer with high density and helical side chains, under the aryl alkyne–Pd(II) catalyst *via* living chain-growth polymerizations. This investigation proved that brush density significantly affected their viscosity, modulus, and performance [61]. Nevertheless, the preparation of Pd(II) catalyst is complicated and costly, which is unsuitable for the industrial-scale synthesis of brush polymers. Therefore, the development of high-performance and environmentally friendly catalysts is crucial for the synthesis of phenyl isocyanide brush polymers.

Currently, ATRP and RAFT polymerization have been used to synthesize various brush polymers, but many issues still need to be addressed, such as gelation, catalyst toxicity, and low graft density. Therefore, for the synthesis of brush polymers, several factors play an important role, including the green, simple, and efficient catalysts and chain transfer agents, as well as achieving precise control for brush arm length and grafted density. Many investigations about the synthesis of bottle brush macromolecules focused mainly on linear side chains. Single block side chains may limit the advantages and applications of brush polymers. Moreover, it is possible to construct branched structures, monocyclic and polycyclic structure on the side chain, which may greatly improve the hydrophilicity and hydrophobicity, as well as charge density, assembly behavior, and load capacity of brush polymers. In addition, the unique structural and functional characteristics of brush polymers make them promising candidates for gene and drug delivery, especially for the responsive release and assembly behavior regulation of brush polymers, allowing for improved control of gene and drug release.

### Preparation of star-shaped polymers

Star polymers are a type of branched polymer structure that consisted of a central core and multiple linear arms radiating from the core. Due to their unique nanostructure, the star polymers showed many distinctive physical and chemical properties, including packaging capabilities, core and arm functionalization, superior gene and drug delivery properties [16, 39]. In 1968, Zilliox and co-workers used multifunctional monomer and initiator (such as isopropyl potassium or butyllithium) to successfully synthesize star macromolecules *via* anionic block polymerization. This approach also were applied to prepare various types of star-shaped macromolecules, including polystyrenes (PS), polyisoprenes, polyvinylpyridines and poly(methyl methacrylate) (PMMA) [15, 16, 62]. The preparation of star polymers can generally be categorized into two main strategies, including arm first and core first. In addition, many methods for preparing star polymer have been summarized in detail, including ROP of cyclic esters, its derivatives, and N-carboxyanhydride, single electron transfer living radical polymerization (SET-LRP), ring-opening metathesis polymerization (ROMP), ATRP, and RAFT [15].

#### Preparation of star-shaped polymers *via* arm first strategy

**Fig. 3:**
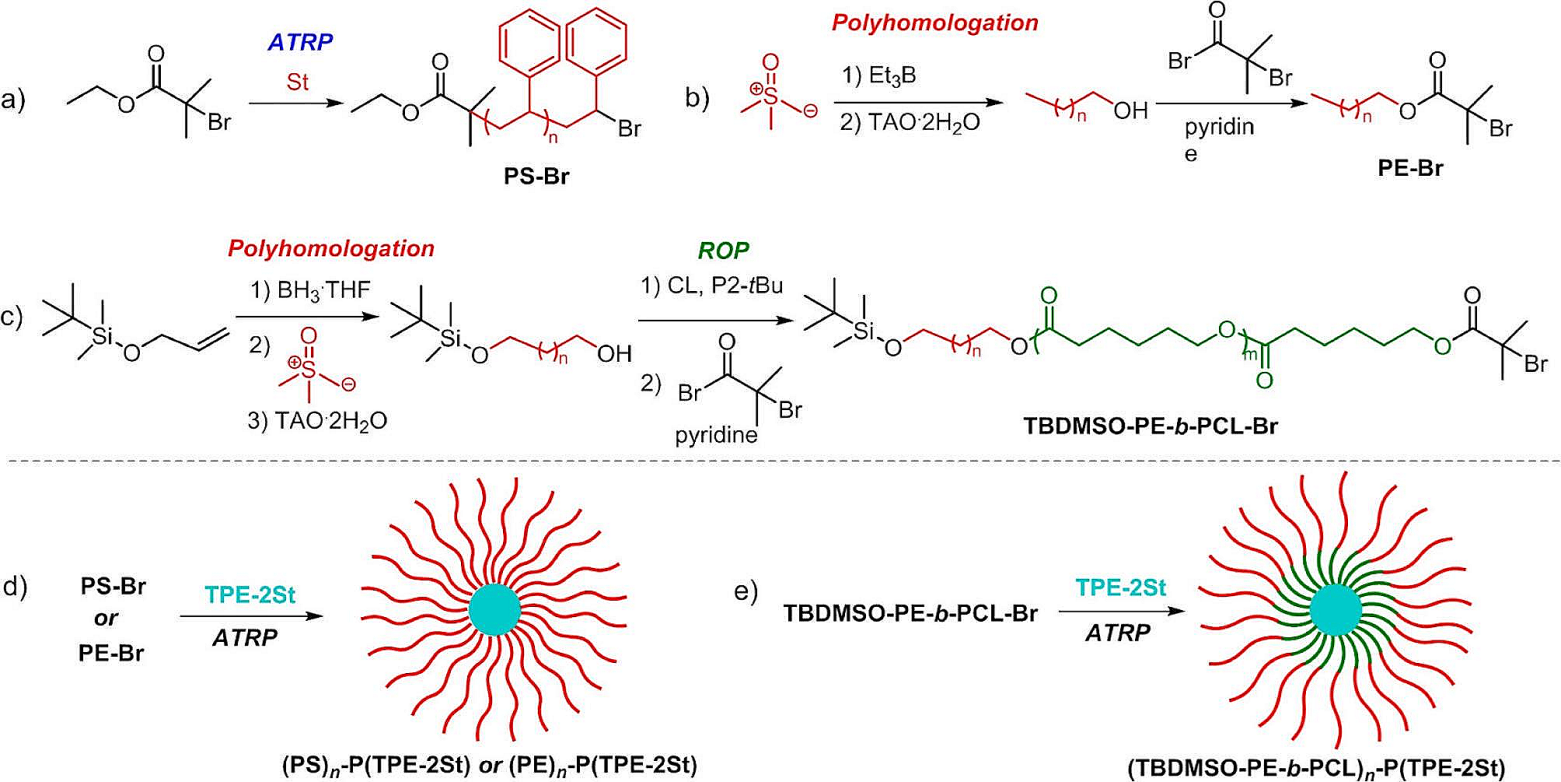
Synthesis of star-shaped polymer *via* arm-first method [64]

Owing to the advantages of arm first strategy for the multi-arm star polymers synthesis, Ding et al. utilized electron transfer ATRP to prepare star polymers with block copolymer arms. Firstly, they prepared poly(butyl acrylate-tert-butyl acrylate) with linear block, and then crosslinked the end groups of linear molecule’s with divinylbenzene to synthesize a multi-arm star block polymer [63]. They were prepared several macroinitiators by activators regenerated by electron transfer (ARGET) ATRP to reactivate the deactivation activator and achieve effective controllable polymerization at the ppm catalyst concentration. The ARGET-ATRP greatly overcomes the complicated separation between polymer and catalyst during the polymerization process. Hadjichristidis et al. employed double styrene-functionalized tetraphenylethene exhibiting aggregation-induced emission (AIE) characteristics as the core, and polystyrene, polyethylene or polyethylene-b-polycaprolactone as arms to construct a multi-arm star polymer through an arm-first approach (70% yield in the two steps) (Fig. 3) [64]. Current research suggested that the AIE characteristics of molecular could be manipulated through topological structure adjustments, which improved the applicability of AIE molecules in dilute solutions. Therefore, topological structure could play a crucial role in the design of advanced responsive materials with enhanced performance and application prospects. Pilkington et al. developed a novel multi-armed star-shaped poly(2-hydroxyethyl acrylate) using an arm-first strategy to reduce the cytotoxicity of human amylin peptides [65]. Moreover, this versatile approach favors the synthesis of multi-component block star polymers. Yoshizaki et al. utilized active cationic polymerization to quickly and quantitatively prepare various star poly(p-methoxystyrene) *via* the arm-first strategy [66]. Firstly, an arm molecule with terminal groups was synthesized. Finally, a network core molecule was formed through cross-linking terminal vinyl groups of the arm molecule. The network structure molecule is a novel star polymer core, which can replace many traditional core molecules. Compared with RDRP, one-step quantitative polymerization can achieve more efficient active cationic polymerization, which provides a new idea for the preparation of specialized star-shaped polymers.

#### Preparation of star-shaped polymer *via* core first strategy

Zhang et al. used polyhedral oligomeric silsesquioxane (POSS) as the core and block PCL-PDMAEMA as the arm to construct a functional amphiphilic block star polymer *via* ROP and RAFT polymerization [67]. The less branched site in POSS affects the mechanical strength, flexibility, and other desired properties, limiting its application. Similarly, Yang et al. reported poly(glycidyl methacrylate) grafted cyclodextrin(CD) as core to prepare star polymers with high gene transfection efficiency and low toxicity *via* core-first strategy [68]. They firstly grafted ATRP initiator to CD, and then used ATRP to synthesize a poly(glycidyl methacrylate) arm. Finally, a series of five-arm responsive and degradable star polymers were synthesized. Due to the reaction sites limitations of core, only fewer arms can be grafted onto the polymer. Thus, increasing the number of branches site of core is a key research for the development of various star polymers.

**Fig. 4:**
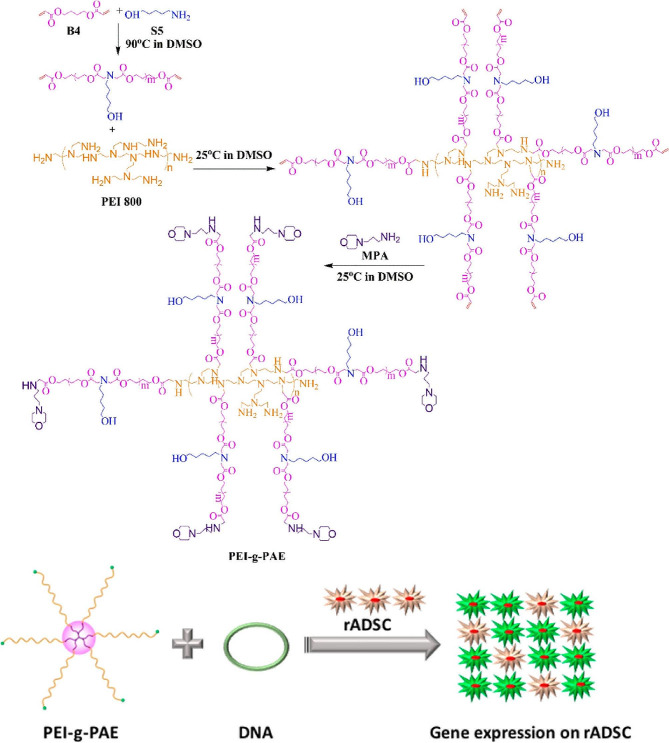
Synthesis of star-shaped polymer *via* core first method [69]

Lam et al. used PAMMA dendrimer as a core to prepare multi-arm star-shaped polymers through ROP of lysine and valine to construct a star-shaped peptide polymer as structural nanoengineered antibacterial peptide polymers (SNAPPs) [70]. Compared with other types of cores, PAMAM dendrimers, as a type of dendritic polymer, have complete symmetry and abundant branched chains, making them advantageous in the preparation process of multi-arm star polymers [71]. Subsequently, Zhou et al. reported a novel star polymer *via* arm first methods for gene delivery vectors [69]. Firstly, linear poly(β-amino ester) (LPAE) was prepared by Michael addition from 5-amino-1-pentanol and 1,4-butanediol diacrylate. Finally, LPAE was reacted with branched polyethyleneimine, and then end-capped with 3-morpholinosydnonie was added to form a star polymer (Fig. 4). Gel permeation chromatography (GPC) result and ^1^H NMR spectrum demonstrated the successful synthesis of the star polymer. This research cleverly combined the advantages of different structures’ polymers (low toxicity and high transfection) to design a new star-shaped polymer gene delivery vehicle. Under the same design concept, Zhou et al. used single-chain internal cyclized/knotted polymer (SCP) as the core and LPAE as the arm to prepare high-performance star polymers through the reaction of sulfhydryl groups and double bonds [72]. Therefore, the combination of multiple structural polymers to prepare various star polymers or other new structural polymers is a simple and efficient method. Currently, there are many available methods for the preparation the star polymers preparation each with its own advantages and limitations. Altogether, arm first strategy is more conducive to the preparation of multi-arm star polymers, while core first strategy is more conducive to the preparation of multifunctional star polymers. For star polymers, the investigation of multifunctional arms, multi-arm arms, and various structural core molecules holds great promise from fully degradable alternative to leading non-degradable polymeric vectors.

### Preparation of branched structure polymers

Due to their only two terminal groups and few functionalized sites, linear structure polymers exhibited poor DNA binding ability and low cellular uptake efficiency. To solve these problems, many non-linear topological structure polymers mainly including linear-branched and hyperbranched polymers were designed. Hyperbranched topological polymers are characterized by multiple terminal groups and broad three-dimensional structures to modify their physical and chemical properties [73–75]. Nevertheless, the increase of terminal groups promotes intermolecular cross-linking, which tends to cause gelation and severely limits the controllability of high MW hyperbranched polymers [5]. We will also summarize the synthesis approaches of several typical hyperbranched polymers including highly branched poly(β-amino ester)s (HPAEs), branched PEI (BPEI), and branched PAMAM.

**Fig. 5:**
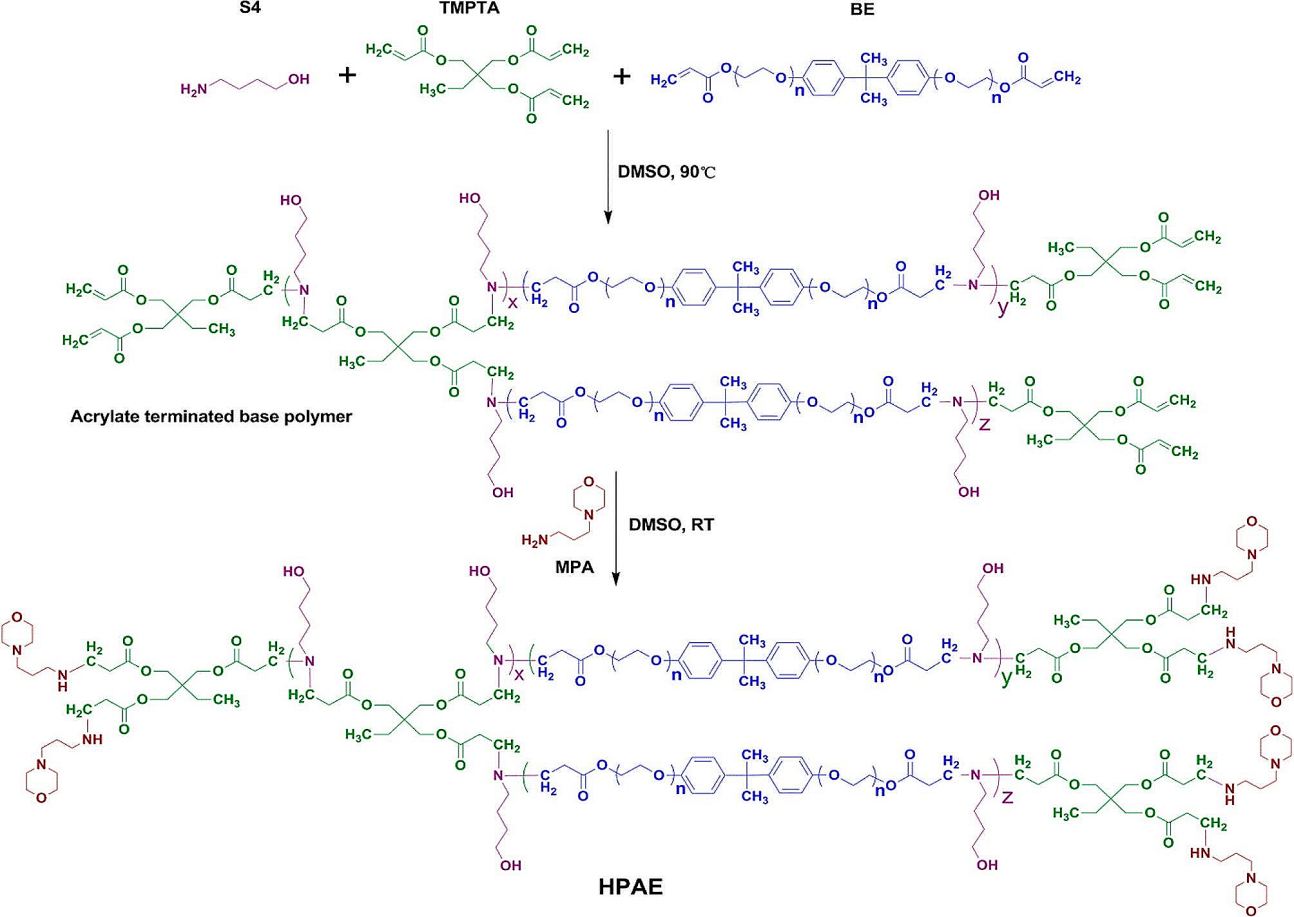
Synthesis of HPAEs *via* an “A2 + B3 + C2” type Michael addition reaction [76]

Many investigations have demonstrated that, BPEI [77], branched PAMAM [78], and branched poly[2-(dimethylamino)ethyl methacrylate] (PDMAEMA) [27], are superior to their corresponding linear counterparts in gene transfection efficiency. In 2015, Zhou et al. used a one-pot “A2 + B3 + C2” type Michael addition approach to synthesize 24 highly branched poly(β-amino esters)s (HPAEs) (Fig. 5), and characterized their structure, purity and compositions through ^1^H NMR spectroscopy and GPC [76]. The “A2 + B3 + C2” Michael addition strategy offers multiple advantages, including 1), the broad accessibility of triacrylates facilitated the efficient and scalable synthesis of HPAEs; 2), the equal reactivity of amine groups and acrylate monomers greatly promoted the formation of HPAEs, and effectively inhibited gelation during the polymerization process [5]. In 2018, Zeng et al. combined the linear oligomer branching strategy to prepare a new linear-branched hybrid poly(β-amino ester) (LBPAE), which provides an important platform for the synthesis of structurally precise branched polymers [79]. Liu et al. designed and optimized a new disulfide in backbone and grafted guanidine moieties to prepare HAPEs *via* iteratively optimizing reaction strategies, exhibited high transfection efficiency in adipose derived stem cells (ADSCs) and astrocytes [4].

BPEI is an efficient gene delivery vector with outstanding gene condensation ability to form stabilized nanoparticles, facilitated endosome escape, and gene transfection due to its high density of amine groups [80]. Nevertheless, the high positive charge induced that BPEI showed high cytotoxicity, which severely limited its clinical application. Therefore, various functionalization of BPEI including block polymerization, the balance of the charge density for terminal groups and hydrophilicity and hydrophobicity have been investigated [81]. To improve its biodegradability, Zhang et al. used disulfide bonds to link adamantane-BPEI and polyaspartamide *via* the host and guest interaction of cyclodextrin and adamantane, to construct a biodegradable nano-delivery supramolecular vectors [82]. Kihoon et al. adopted disulfide bond-containing molecules as the core to prepare BPEI through repeated Michael addition-amidation reactions, which greatly reduced its cytotoxicity after transfection [83]. More importantly, this study provides a generalized platform for branched polymer synthesis.

**Fig. 6:**
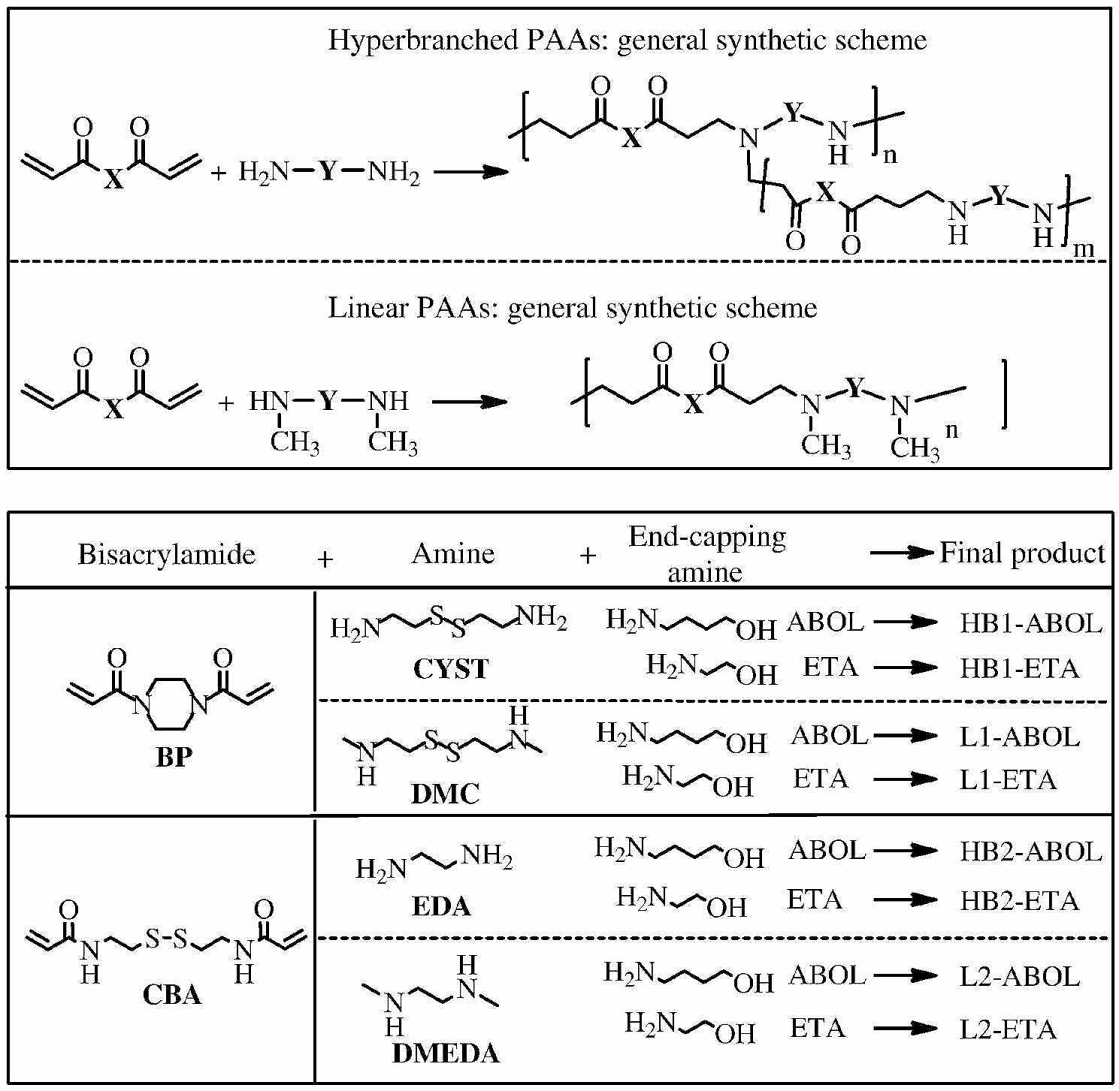
Synthesis of functionalized hyperbranched PAA *via* Michael addition reaction [84]

Martello et al. used diacrylamide and diamine as monomers to prepare a series of hyperbranched PAMAM through Michael-type polyaddition of the appropriate bisacrylamides and amines for responsive degradation (Fig. 6). In addition, GPC and ^1^H NMR spectroscopy characterized all hyperbranched PAMAM. Transfection results illustrated that hyperbranched polymers showed higher gene transfection efficiency than linear PAMAM duo to advantages of hyperbranched topology [84]. Furthermore, the versatile method may be employed in the synthesis of other polymers with different monomer composition and structure, branching degree and MW.

In addition, a series of functional degradable branched PDMAEMAs were prepared *via* controlling the crosslinking copolymerization of vinyl monomer. Hyperbranched PDMAEMA was prepared *via* in situ deactivation enhanced ATRP, and then the double bond was end-capped by N-(3-Aminopropyl) morpholine by Michael addition to effectively inhibit gelation [27]. For preparing multi-component branched PDMAEMA, the new method could be applied to the block copolymerization from other vinyl monomers.

Therefore, the development of hyperbranched polymers may focus on the following aspects, including, (1) developing precise design and preparation methods of hyperbranched polymers; (2) grafting multifunctional molecules at terminal hyperbranched structures to regulate self-assembly behavior, biodegradation, targeting, controlled release and other characteristics; (3) developing the complex structured polymers combined hyperbranched structures and other structures; (4) exploring the relationship between branching structure and application performance.

## Recent application research of non-linear topological polymers in gene therapy

**Fig. 7:**
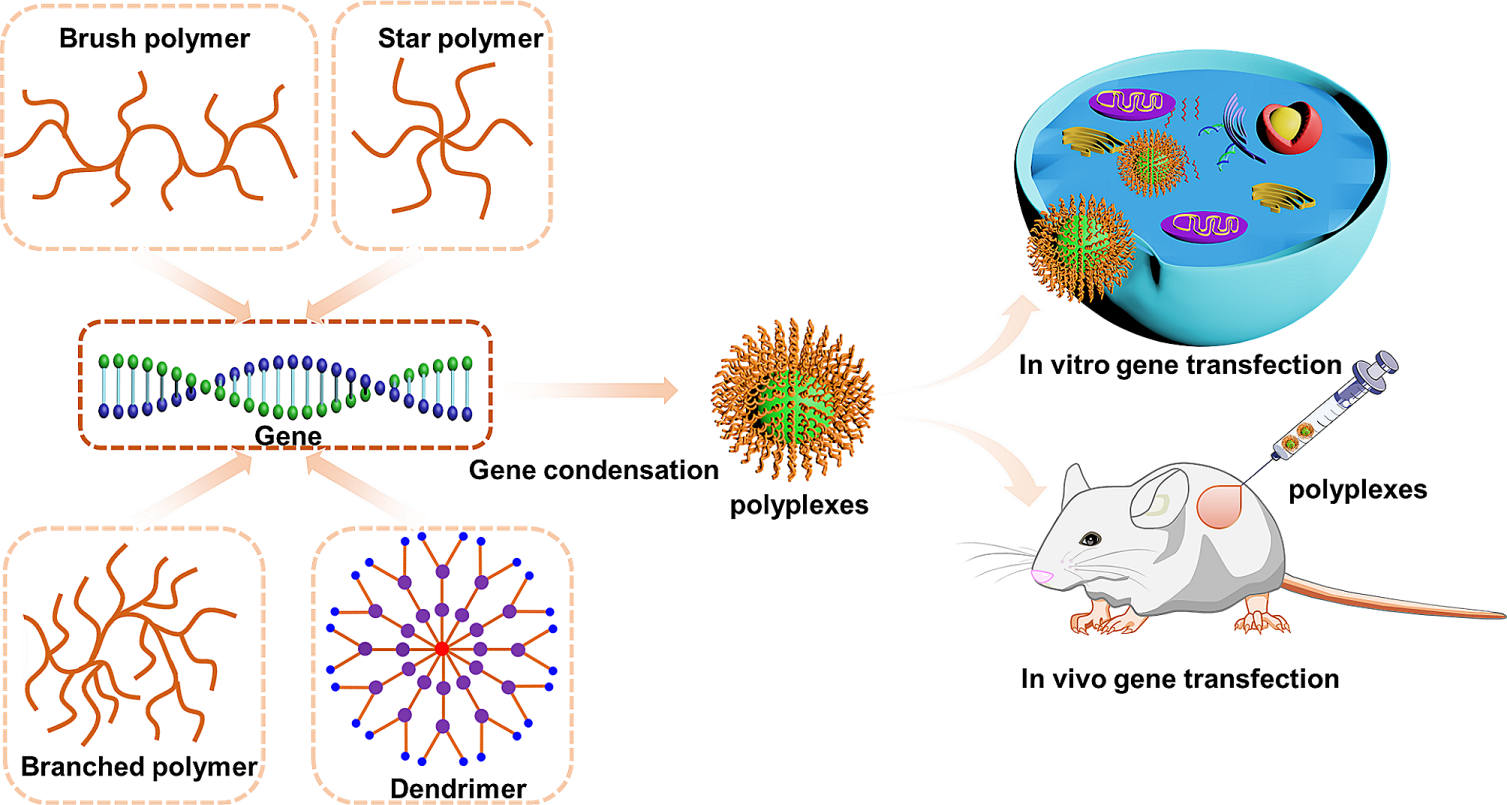
Non-linear topological structure polymers for gene transfection in vitro and in vivo

Almost all human diseases are related to genes, gene therapy such as replacing faulty genes or silencing mutant genes may become a potential therapeutic strategies for many diseases [85]. Gene therapy is the process of introducing genetic material into specific cells to achieve the purpose of treating diseases by replacing wrong gene expression or inhibiting specific gene expression, such as hemophilia, muscular dystrophy, cystic fibrosis, neurodegenerative diseases, diabetic wound healing, recessive dystrophic epidermolysis bullosa [85–88]. Nevertheless, efficient gene delivery need to be overcome several barriers, mainly including: the extracellular barriers, nuclease degradation and protein adhesion in serum, clearance by phagocytic cells, electrostatic repulsion, and intracellular barriers, endosome degradation, transport barriers of proteins and microtubules in the cytoplasm, and the effective release of genetic material [89–91]. Therefore, gene vectors need to be characterized with various superior properties, including, high DNA condensation ability, strong charge density, high serum stability to prevent protein accumulation and endonuclease degradation, specific targeting and cellular uptake, efficient endosomal escape, efficient transport in the cytoplasm, DNA release at a certain time, and nuclear localization for transcription [1, 92–94].

However, due to the complicated topological structure, multiple functional modification and diversified composition, non-linear topological structure polymers in gene delivery hold great prospects (Fig. 7). Due to the advantages of topological effects, various non-linear topologically structured polymers have demonstrated exceptional DNA, mRNA, siRNA delivery properties. Additionally, there are numerous intriguing findings highlighting the non-linear topology polymers in gene delivery, including brush polymers, star polymers hyperbranched polymers, and dendrimers. This chapter aims to provide an in-depth exploration of the progress of various non-linear topology polymers for gene therapy in recent years.

### The current status of brush polymers in gene therapy

**Fig. 8:**
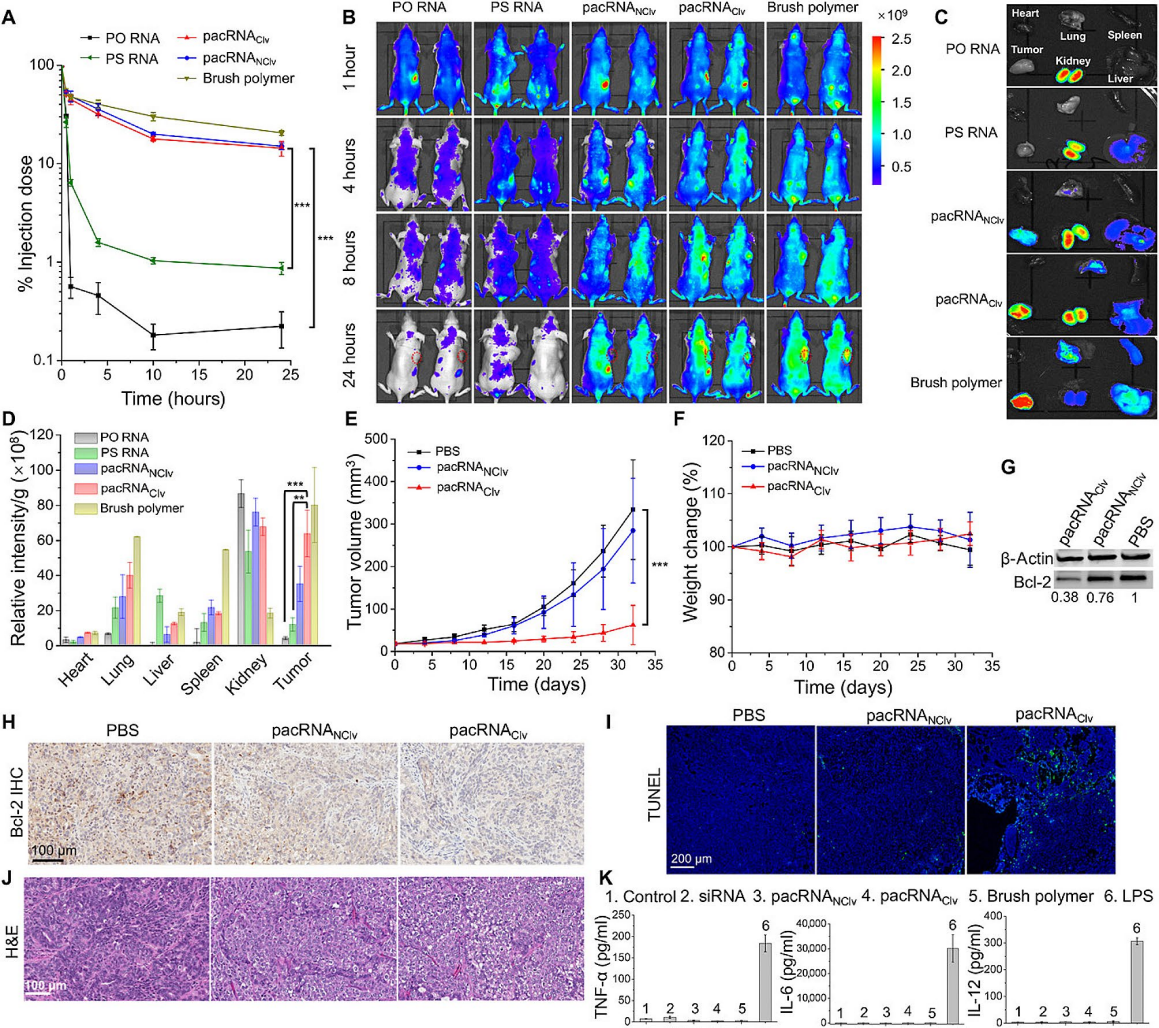
PEG-based brush polymer efficiently mediates gene silencing, reduce strong anti-tumor effect [97]

Compared with linear polymers, the brush polymer with more side chains, exhibited higher DNA condensation and gene transfection efficiency. Zhang et al. used ATRP to synthesize a new type of star-shaped brush polymer, showing higher transfection efficiency in vitro than star polymers and PEI [95]. This study suggested that the topological effect and branching degree of polymers significantly affect their DNA transfection efficiency. By understanding the internal relationships between polymeric structure and transfection efficiency, more effective gene delivery systems could be designed. The weak electrostatic interaction between DNA and vectors can lead to premature DNA leakage, limited the subsequent transfection [96]. Thus, many responsive releases were reported through chemical bond breaks or topological structure changes to modulate the interaction between the vector and DNA. Wang et al. demonstrated that the PEG-based brush polymer with disulfide bonds could mediate targeted release of siRNA for cancer cells (Fig. 8)[97]. Furthermore, the cellular uptake, in vitro transfection efficiency and post-transfection cellular viability suggested that the brush-like structure enhanced the nuclease stability cellular uptake, and siRNA delivery. Notably, its blood elimination half-life was increased 19-fold. The anti-tumor efficiency and safety in vivo strongly supported the PEG-bottle brush polymers as an effective long-circulating vector for siRNA silencing therapeutics. Blum et al. demonstrated that the density of cell penetrating peptides arm significantly affects the gene editing efficiency of brush polymers, highlighting that functionalized arms effectively facilitate their gene and protein delivery [44]. Ahern et al. also demonstrated that the charge density and hydrophobicity of the arms influenced polymer-mediated transfection efficiency and cytotoxicity [98]. Due to good biocompatibility of the natural materials, Nie et al. grafted polymethacrylic acid onto heparin’s side chain to prepare natural based brush polymers for gene delivery [99]. The transfected results in vitro proved that the brush polymer (PDMAEMA-heparin) mediated higher transfection efficiency than that of PEI. Their results preliminarily showed that heparin-based brush polymers can be used as potential vectors, but lack of more systematic validation in animal testing, and demonstrated that non-linear topological structures polymers from natural polymer backbones or side chains have important potential in gene delivery. Similarly, in vitro and in vivo data also suggested that brush-shaped and sun-shaped PDMAEMA exhibited lower cytotoxicity and higher transfection efficiency, particularly for difficult-to-transfect primary human T cells [14]. Compared to linear analogues, brush polymers and sun-shape polymers possessed more side chains, which reduced the non-specific interaction between vectors and cell membrane, reducing the rupture of cell membranes and facilitating gene transfection [100]. Thus, multiple modifications for side chains can endow brush polymers with many properties to meet the multiple requirements of gene delivery, including cytotoxicity, endosomal escape, and nuclear localization.

In contrast, there are some drawbacks. Brush-structured polymers, due to their random fragments, exhibited slightly poor packaging property and colloidal stability, limiting their transfection efficiency. Nevertheless, the main chain for sun-shaped polymer is looped, resulting in more stable performance. The formation of the primary backbone reduces the hindrance between the side chains, therefore the polymer possessing a sunflower-like or star structure polymers may offer greater advantages.

### The current status of star polymers in gene therapy

Due to their well-defined structure, flexible modification, higher transfection efficiency, the star polymers were widely used for gene delivery. The researches mainly include multifunctional arms, hydrophilic modification, and CD as cores [101, 102]. However, there are few studies on the balance of hydrophilicity, charge properties and density for integration of diagnosis and treatment. Most of the star-shaped polymer vector is still in the preliminary stage lack of in-depth systematic research.

#### Star polymers for siRNA delivery

Through in vitro experiments, Cho et al. preliminarily proved that PEG-armed star polymer could highly efficiently deliver DNA and siRNA in S2 cells [103]. Li et al. demonstrated that the poly(oligoethylene glycol) methacrylate-cationic hyperbranched polymer showed superior biocompatibility and less steric hindrance to enhance their siRNA binding capacity and gene silencing efficiency, indicating that brush cationic polymers show significant advantages for gene silencing to treat heterotopic ossification [104]. Nevertheless, there are few reports on star-shaped or sun-shaped polymers with hyperbranched polymers as the core for gene delivery. Therefore, this investigation provided a valuable reference for the gene delivery of multiple hyperbranched polymers and star polymers. Furthermore, owing to the biocompatible polypeptide and PEG arms, a polypeptide–PEG miktoarm star copolymer mediated high cellular uptake efficiency and transfection efficiency, low cytotoxicity in A549 cells [105]. The transfection results indicated that the miktoarm star copolymer-mediated luciferase gene silencing efficiency was more than 68% at a siRNA dose of 150 nM. Based on its fluorescent property, the star polymer with polypeptide-PEG miktoarm could serve as a probe for intracellular transport pathway, which may meet both gene delivery and bioimaging, and the integration of diagnosis and treatment.

**Fig. 9:**
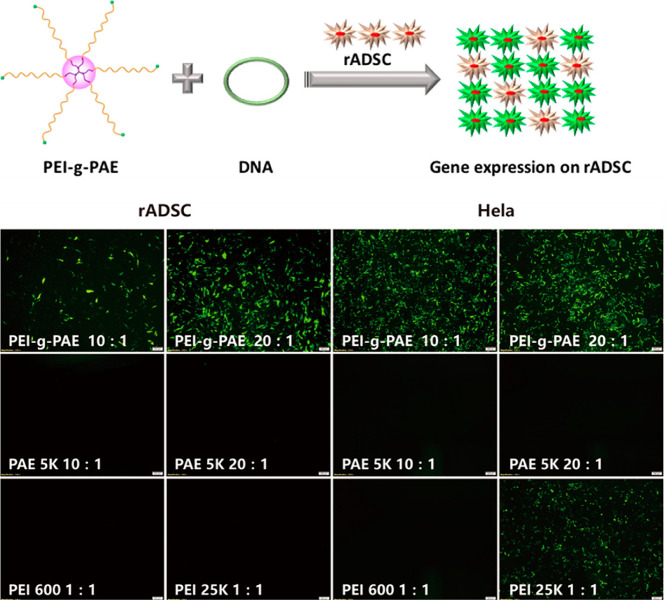
The new star-PAE exhibited high gene transfection efficacy in rADSC and HeLa cells [69]

#### Star polymers for DNA and mRNA delivery

Due to the advantages of amine groups, phosphate groups, star topology structure, star polymers with PDMAEMA arms showed low cytotoxicity and high transfection efficiency in vitro [106]. A star polymer with P(DMAEMA-co-OEGMA-OH) arm was firstly used to efficiently deliver DNA and mRNA, providing a valuable insight for the development of delivery systems for multiple genetic materials [107]. Considering the performance advantages of PEI and PAE in gene delivery, Huang et al. used grafting-onto approach to synthesize a new star-shape PAE polymer consisting of low molecular weight PEI as a core, and low molecular weight LPAE as arms. More importantly, the optimized star-shape PAEs achieves superior gene transfection efficiency, and low cytotoxicity, which were 264-fold and 14,781-fold higher gene transfection efficiency of ADSC than that of the individual PEI and LPAE, respectively (Fig. 9) [69].

**Fig. 10:**
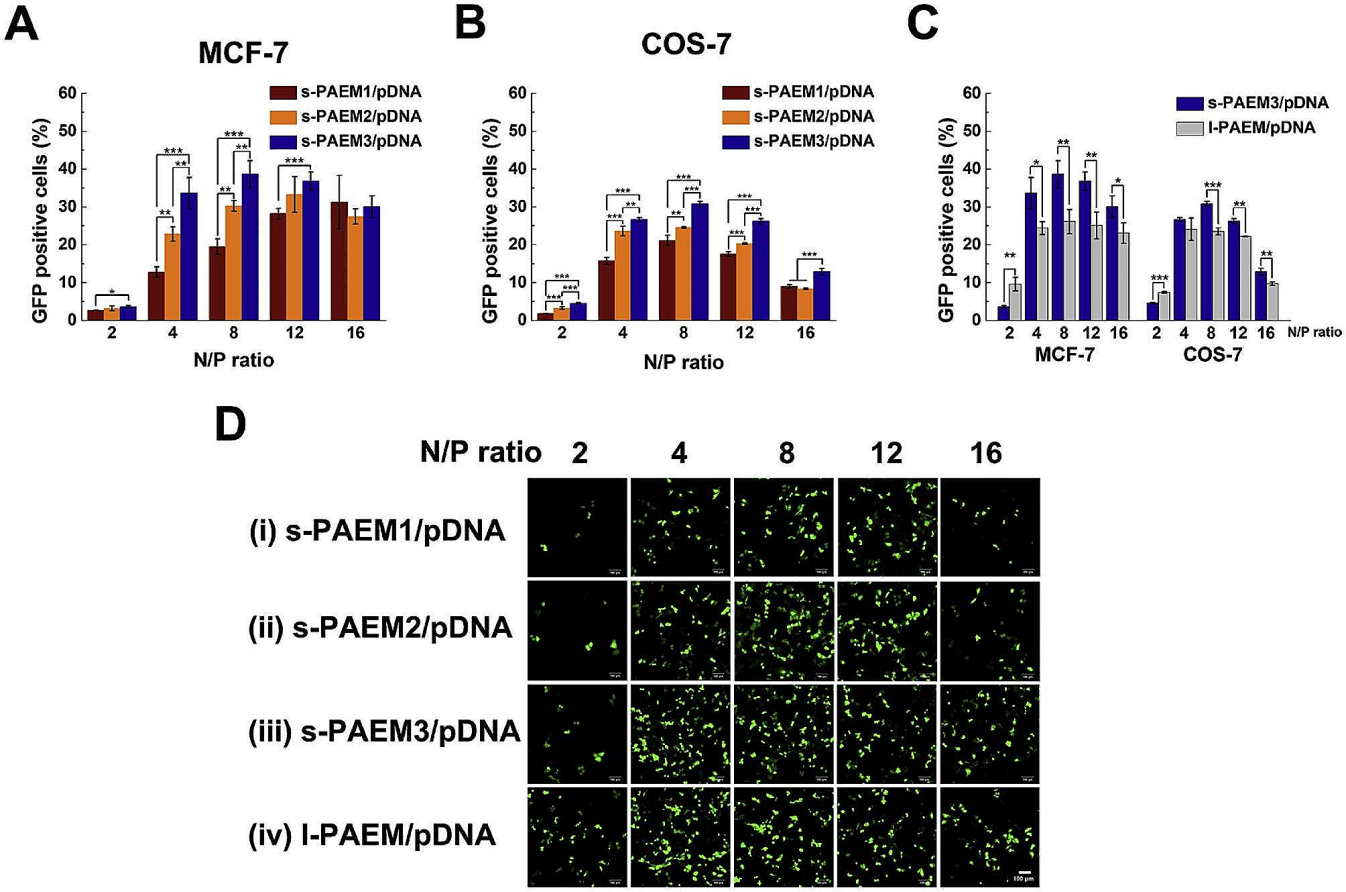
Three star polymers mediated high GFP transfection efficiency in MCF-7 and COS-7cells [108]

In 2019, Zhang et al. systematically explored relationship between star structure and gene delivery property [108]. These findings demonstrated that the star-shaped structure showed less effect on the DNA condensation, while significant effect on its transfection efficiency and cellular activity (Fig. 10). At the N/P ratio of 2 to 12, the transfection efficiency of star-shaped polycations was higher in MCF-7 cells than in COS-7 cells, which initially illustrated the relationship between the star-shaped structure and the mechanism of the transfection step.

Understanding the relationship between polymer structure and each step of the transfection process is crucial for optimizing gene delivery efficiency of polymer. Therefore, it is indispensable to further investigate the specific mechanisms involved in each step of the transfection process to develop and design of high-performance systematic polymer vectors. In addition, silicon-γ-Fe_2_O_3_ as the core with star polymer exhibited high gene transfection efficiency for difficult-to-transfect cells [109]. The preliminary research also greatly expands inorganic nonmetal/metal nanoparticles as the core to synthesize star polymer for difficult-to-transfect cells gene delivery.

Henceforth, forthcoming investigations concerning star polymer vectors will primarily encompass: 1). Design of various core with numerous active sites, visualizability, and biodegradability; 2). Exploration of diverse architectures and multifunctional arms, especially for precise hybrid arms; 3). Development of composite structural system of star polymers and other topological structural polymers. Owing to the limitation of the one core, the development of multi-core structures in star polymers may have potential to meet the requirements of ideal gene vehicles. Hyperbranched polymers, similar to star polymers, possessed numerous branches from many cores.

### The current status of branched polymers in gene therapy

Due to their multiple terminal groups and 3D space structure, branched polymers showed many distinctive performances. Furthermore, many studies have demonstrated that branched PEI, PAMAM, PDMAEA, PAE, CS, PLL have higher transfection efficiency in gene delivery compared to their linear counterparts [1, 5, 110]. Currently, The HPAE are promising nonviral vectors for various functional gene delivery and potential use in gene therapies.

#### The current status of HPAEs in gene therapy

**Fig. 11:**
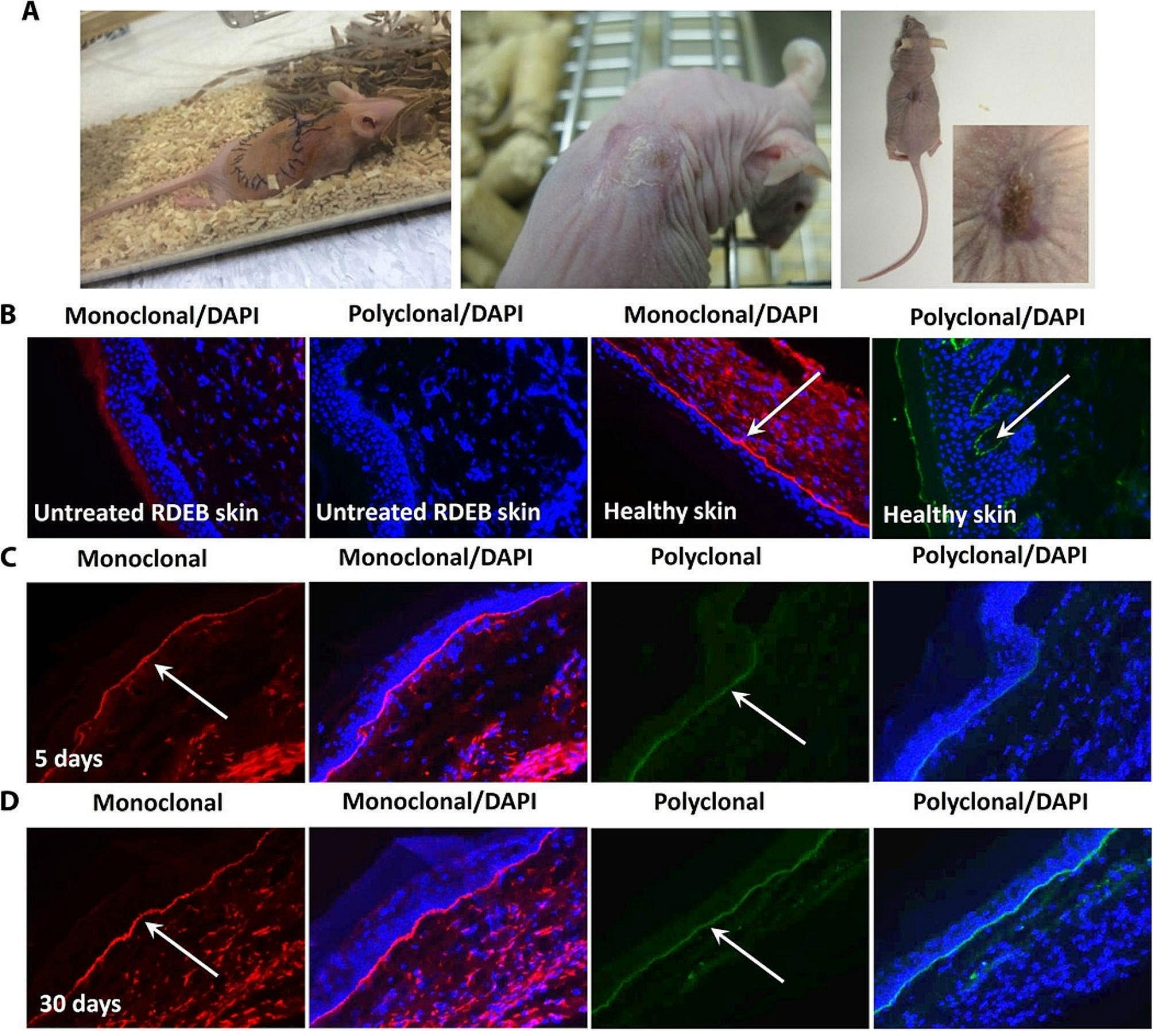
Optimized HPAEs showed great superiority in gene transfection in vivo [5]

In 2000, Langer et al. firstly prepared linear poly(β-amino ester) (LPAE) *via* the “A2 + B2” Michael addition reaction of diamine and diacrylate monomers, which was considered to be one of the most effective non-viral gene delivery vehicles [111]. In 2015, Zhou et al. developed a “A2 + B3 + C2” Michael addition reaction to synthesize HPAEs for gene delivery, and preliminarily evaluated the biophysical properties and transfection performance, and finally reported that HAPEs exhibited ultra-high transfection efficiency for skin-related cells [76, 112]. More importantly, the results indicated that the optimized HPAEs showed up to 8521-fold in gene transfection efficiency compared with the corresponding LPAEs and lower cytotoxicity in 12 types of cells including primary cells, stem cells and nerve cells [5]. Results from animal experiments have shown that optimized HPAEs can effectively deliver DNA encoding collagen VII (C7) into keratinocytes and fibroblasts, and restore C7 expression and secretion for 30 days (Fig. 11), which indicated branched structures can enhance the transfection efficiency and safety of HPAEs, highlighting the great potential for the successful application of non-viral gene therapy in inherited skin diseases [5, 113].

To further explore the relationship between linear and branched architecture, Zhou et al. used branched monomers linking oligomeric LPAEs to prepare a new type branched-linear poly(β-amino ester)s (H-LPAEs) for gene delivery [79]. They systematically evaluated the biophysical properties of H-LPAEs, including proton buffer capacity, DNA condensation, DNA release, cellular uptake. In human primary dermal fibroblasts (HPDFs) and mouse embryo fibroblasts experiments show that the H-LPAEs demonstrated superior transfection ability, revealing the advantages of branching for polymer gene delivery. Zhao et al. synthesized a new structure of linear-branched poly(β-amino ester)s (H-LPAEs) through the linear oligomer combination branched strategy, also demonstrated that optimal H-LPAEs mediated 56.7% and 28.1% cell apoptosis in HepG2 cells and HeLa cells, which highlighting its potential application in cancer gene therapy [114].

In 2020, Wang et al., prepared a series of HPAEs *via* “A2 + B3 + C2” and “A2 + B4 + C2” strategies, and confirmed that branching strategies showed a significant impact on DNA condensation, cell uptake, and DNA transfection [115]. Meanwhile, various functionalized modifications of HPAEs, such as responsive degradation, cancer cell targeting [116], pH and temperature responsive release [117], the balance of hydrophilicity and hydrophobicity [118], guanidine moiety modifications [4] have been employed for the genetic material delivery, especially for difficult-to-transfect cells. Compared to the limitations of single functionalization, iterative optimization could comprehensively improve the transfection performance of HPAEs, which becomes an important strategy for the optimized multifunctional polymer-based vectors [4]. Nevertheless, the spatial obstacles and performance differences for multiple functional monomers may cause many unfavorable restrictions. Therefore, the development of multifunctional monomer may play a key for future modification. In general, the majority of investigations focus on gene delivery in vitro and in vivo, lacking systematic clinical application. Therefore, based on the existing research foundation, systematic clinical trial studies are imperative for the development and expansion of HPAE-based polymeric gene vectors.

#### The current status of other hyperbranched polymers in gene therapy

**Fig. 12:**
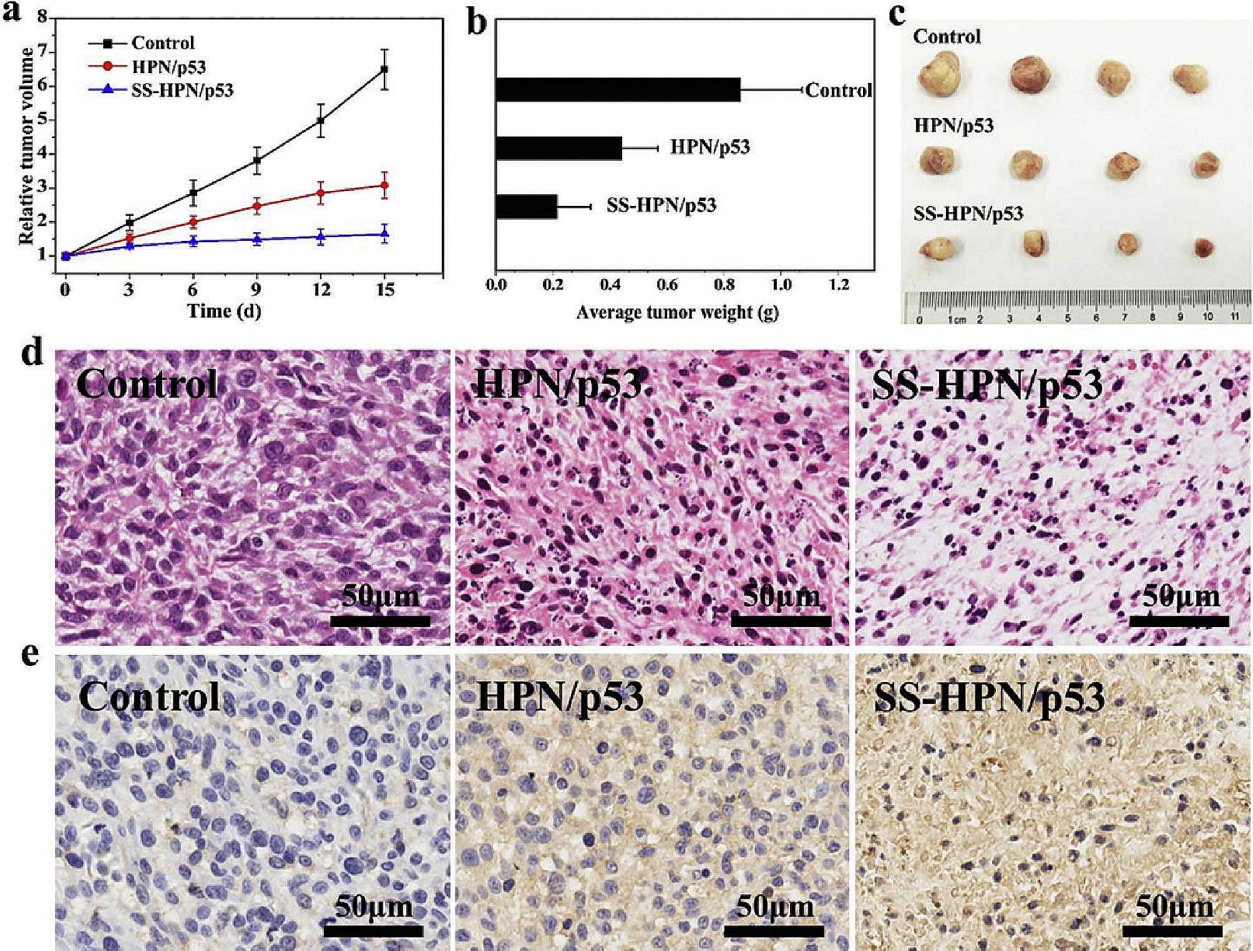
Transfection and tumor suppression effect of hyperbranched polyaminoglycosides [120]

To improve the charge density, targeting and biodegradability of branched PAMAM, Gu et al. prepared a multifunctional composited branched PAMAM nanoparticle. The quantitative results proved that the gene expression of the multifunctional branched PAMAM/DNA nanoparticles were approximately 4.5, 6.3, and 2.3-fold higher compared with the Lipofectamine/DNA complexes, where the gene silencing efficiency was up to 71% after nanoparticle transfection, which effectively inhibited the growth of CD44-positive tumours [119]. Inspired by the application of a new generation antibacterial, drug/gene delivery biomaterials, Huang et al. prepared multifunctional hyperbranched polyaminoglycosides with disulfide bonds, and demonstrated that multifunctional hyperbranched polymer exhibited superior antibacterial and biocompatibility, and lower cytotoxicity than that of PEI at all weight ratios in HEK 293 cells (Fig. 12) [120]. In vitro gene transfection results indicated that the degradable hyperbranched polyaminoglycosides could achieve efficient *P53* DNA delivery and inhibit tumor growth to facilitate the development of next-generation biomaterials.

To improve the therapeutic efficacy of nasopharyngeal carcinoma, Liu et al. constructed a degradable BPAA based vector with functionalized graphene oxide for transferrin-targeted drug/gene co-delivery, and showed higher antitumor effect in vivo [121]. Their investigations provided a facile platform to integrate the drug/gene co-delivery strategy and targeting effect into one nanoparticle for the nasopharyngeal carcinoma therapy. To reduce the cellular toxicity of hyperbranched PEI (HPEI), Cook et al. prepared a novel of hyperbranched poly(ethylenimine-co-oxazoline) *via* thiol-yne reaction and acid hydrolysis [74]. In vitro experiments proved that the block HPEI showed lower cytotoxicity, but the transfection efficiency was slightly lower than the commercial standard BPEI. In addition, Guo et al. grafted polyglycine to branched PEI, and then compounded with poly(ethylene glycol)-poly-L-glutamate to construct a new PEI-based polymer vector for DNA and siRNA delivery [122]. The studies demonstrated that the PEI-based gene vector not only exhibited superior DNA and siRNA transfection capacity in various cell lines, but also had lower cell toxicity compared with PEI.

Numerous investigations have illustrated that hyperbranched polymers have many significant advantages in gene therapy. However, there are still many shortcomings, such as poor structural accuracy and many preliminary studies, which hinder their clinical applicability. Undoubtedly, it is very meaningful to undertake a comprehensive exploration of hyperbranched polymer vectors from three aspects. Firstly, precise synthesis of diverse functionalized hyperbranched polymers. Secondly, the development of an organic-inorganic hyperbranched polymer nano-delivery system. Thirdly, the conduction of elaborate animal and pre-clinical experiments to uncover profound insights.

#### The current status of dendrimers in gene therapy

Due to their unique characteristics such as precise topology, uniform particle size (monodispersity), and multiple functional terminal groups, dendrimers have gained significant definition in gene delivery [32]. These properties allowed dendrimers to effectively interact with genes and facilitate their efficient delivery into target cells. However, their poor degradation properties often induce higher cytotoxicity, limiting safe and efficient gene delivery. Fattal et al. demonstrated phosphorus-based dendrimers with either pyrrolidinium or morpholinium as terminal protonated amino groups showed good degradability, strong siRNA binding ability, high cellular uptake and silencing efficiency in vitro [123]. These findings suggested that phosphorus dendrimers have great promise in enhancing siRNA delivery for treating lung inflammation. Parat et al. revealed that asymmetric arginine dendrimer with 16 primary amines can facilitate gene delivery in caveolae cells [124]. Due to the high encapsulation efficiency of Au nanoparticles and the excellent biocompatibility of PLL dendrimer, Yu et al. used chitosan grafted PLL dendrimer as a substrate to assemble Au nanoparticles, and surface functionalized cyclized RGD to achieve the integration of tumor imaging and gene therapy [125], which introduced a novel strategy to fabricate PLL dendrimer based nanoparticles for highly efficient cancer diagnosis and therapy.

In addition to multi-terminal non-linear polymeric gene vehicles, there are also terminal-free or multi-cyclic polymers that showed a broad potential for gene delivery in vitro and in vivo [126]. Cheng et al. confirmed that sun-shaped PDMAEMA exhibited higher transfection efficiency than that of comb polymer [100]. In 2012, Wang et al. firstly demonstrated that single-chain structure can increase the DNA condensation to enhance DNA transfection efficiency [127]. To reduce the cytotoxicity of the SCPs, Wang et al. utilized ring-opening addition and RAFT polymerization to prepare SCKP with main chain containing disulfide, indicating that compared to non-degradable polymers, the main chain degradable SCPs exhibited higher transfection efficiency and lower cytotoxicity [128]. In 2013, Wei et al. initially suggested that the cyclic polymer showed higher transfection efficiency and lower cytotoxicity compared to that of the linear counterpart [129]. Similarly, in 2022, due to the compact advantages of the cyclic structure, our investigation also indicated that the cyclic poly(β-amino ester)s exhibited superior gene transfection efficiency and safety profile compared to its linear counterparts [10]. In 2023, multi-cyclic poly(β-amino ester)s also showed higher the transgene expression efficiency compared to their branched counterparts, and were applied to efficiently deliver a CRISPR plasmid for gene editing therapy [126]. These investigations provided a new insight into the relationship between cyclic or multi-cyclic marchitecture of polymer and gene delivery capability, which will strongly stimulate the design and development of a new-generation gene vectors.

## The future development direction of non-linear topological structure polymer gene vectors

Due to multiple end groups, broad and sophisticated spatial architecture and multi-functionality sites, non-linear topological structure polymers including brush-shaped, star-shaped, branched and dendritic structures polymer, offered great promise to deliver functional genes from targeted cells to tissues. However, there are still many challenges for the synthesis and gene delivery of non-linear topological polymers.

Firstly, non-linear topological polymers are difficult to synthesize accurately, mainly relying on the design of special structures of chain initiators or special reaction. For example, star polymers are prone to have fewer arms due to the limited number of reaction sites in the core. Therefore, flexible and controllable design of reaction sites in the core is crucial for the development of multi-functionalized star polymer-based gene vectors. In addition, the chain ends were cross-linked to generate the star or brush polymers with broad MWD, even gelation, which can be unfavorable to achieve polymers.

Secondly, the polymer synthesis process is prone to by-products, nevertheless, the efficient separation of polymers with different topologies still holds a huge challenge, which therefore inevitably limits the synthesis of high-purity polymers with specific topologies. Small amounts of cyclic structured polymers are often generated during the synthesis of branched polymers, which affected the homogeneity of their properties and subsequent transfection efficiency. Therefore, the development of tailored columns or separation methods based on the characteristics of different topological polymers, such as aggregation state or kinetic volume of hydration, is an important fundamental guarantee of the purity of polymers with non-linear topological structure.

Thirdly, dendrimers, as perfectly symmetric spherical and actinomorphous polymers, have demonstrated superior transfection performance. Currently, the synthesis of PAMAM dendrimer is labor-consuming, requiring multiple iterative reactions and protection/de-protection steps, which limits the flexibility and large-scale applications. In contrast to dendrimers, branched polymers such as HPAEs offered a simpler and more efficient synthesis process, exhibited remarkable efficiency, and were easy to produce on a large scale, making branched polymers, especially HPAEs, a promising non-viral gene vector candidate for clinical applications.

Finally, majority of nonlinear topological polymers presently exhibited a relatively single structure, thereby the inherent advantages of topological effects in gene delivery have not been fully exploited. The continued development of diverse novel topologies of polymers such as ring-brush polymers, star-brush polymers, hyperbranched star polymers, and hyperbranched multicyclic polymers, is crucial for the advancement of gene delivery techniques. By exploring the intrinsic relationship between polymer topology and transfection performance, the safer and more efficient next-generation gene delivery vectors may be achievable.

## Data Availability

Not applicable.
